# Diagnosis and Management of Central Congenital Hypothyroidism

**DOI:** 10.3389/fendo.2021.686317

**Published:** 2021-09-09

**Authors:** Peter Lauffer, Nitash Zwaveling-Soonawala, Jolanda C. Naafs, Anita Boelen, A. S. Paul van Trotsenburg

**Affiliations:** ^1^Emma Children’s Hospital, Amsterdam University Medical Centers (UMC), Department of Pediatric Endocrinology, University of Amsterdam, Amsterdam, Netherlands; ^2^Endocrine Laboratory, Amsterdam UMC, University of Amsterdam, Amsterdam, Netherlands

**Keywords:** central congenital hypothyroidism, isolated central congenital hypothyroidism, combined pituitary hormone deficiencies, etiology, diagnosis, management, pituitary stalk interruption syndrome

## Abstract

Central congenital hypothyroidism (CH) is defined as thyroid hormone (TH) deficiency at birth due to insufficient stimulation by the pituitary of the thyroid gland. The incidence of central CH is currently estimated at around 1:13,000. Central CH may occur in isolation, but in the majority of cases (60%) it is part of combined pituitary hormone deficiencies (CPHD). In recent years several novel genetic causes of isolated central CH have been discovered (*IGSF1*, *TBL1X*, *IRS4*), and up to 90% of isolated central CH cases can be genetically explained. For CPHD the etiology usually remains unknown, although pituitary stalk interruption syndrome does seem to be the most common anatomic pituitary malformation associated with CPHD. Recent studies have shown that central CH is a more severe condition than previously thought, and that early detection and treatment leads to good neurodevelopmental outcome. However, in the neonatal period the clinical diagnosis is often missed despite hospital admission because of feeding problems, hypoglycemia and prolonged jaundice. This review provides an update on the etiology and prognosis of central CH, and a practical approach to diagnosis and management of this intriguing condition.

## Introduction

Congenital hypothyroidism (CH) is defined as thyroid hormone (TH) deficiency at birth, either due to defective thyroid gland development or function (primary or thyroidal CH), or due to insufficient stimulation by the pituitary of an otherwise normal thyroid gland (central CH) (1). Central CH is often accompanied by other pituitary hormone deficiencies (combined pituitary hormone deficiency, CPHD), but can also be an isolated condition. Because TH deficiency early in life is harmful to brain growth and development, and difficult to recognize shortly after birth, newborn screening (NBS) programs for CH have been implemented in many countries worldwide since the 1970s. These programs enable early detection and treatment of CH, and successfully prevent brain damage and subsequent mental retardation (2).

The first NBS programs for CH were total thyroxine (T4)-based, combined with, or followed by thyrotropin (thyroid stimulating hormone, TSH) measurement (T4+TSH and T4-reflex TSH, respectively). Although the main objective of these programs was detection of primary CH, they also detected central CH (2). In T4-based NBS, primary CH is suspected when T4 is low in combination with an elevated TSH concentration, and suspicion of central CH arises when T4 is low in combination with a normal TSH concentration. Unfortunately, fairly common but innocent thyroxine-binding globulin (TBG) deficiency also causes a low total T4 in combination with a normal TSH, resulting in a substantial number of false-positive referrals (3, 4). Therefore, many NBS programs switched to, or started with a TSH-based approach which only detects primary CH (5). In support of this, the general notion was that central CH is a rare and often mild condition, and that most neonates with central CH as part of CPHD are diagnosed clinically shortly after birth.

Nowadays only a few NBS programs aim at detection of both forms of CH: several state or regional programs in Italy, Japan, Spain and the USA, and the national programs of Israel and The Netherlands (2, 6–12). **Table 1** shows the characteristics of these programs. In Japan, the number of “false-positives” was reduced by measuring free T4 (FT4) and TSH. In The Netherlands a TBG measurement was added to the existing T4-reflex TSH approach (11, 13, 14) resulting in a three-step process in which T4 is measured in all newborns, and TSH and TBG in those with a T4 concentration belonging to the lowest 20% and 5%, respectively.

**Table 1:** Characteristics of NBS programs screening for both primary and central CH.

Country	NBS approach	Period after birth during which NBS is performed	T4, FT4 or T4/TBG ratio referral rules	Reference
Italy (regional program)	T4+TSH simultaneously	3-5 days of life	Referral if T4 <40 nmol/L	(6)
Japan (regional program)	FT4+TSH simultaneously	4-6 days of life	Referral if FT4 <0.5 ng/dL; second heel prick if FT4 <1.0 ng/dL, then referral if FT4 <1.0 ng/dL	(7)
Spain (regional program)	T4+TSH simultaneously	At 48 hours of life	Referral if T4 <6 μg/dL	(8)
USA (various regional/state programs)	Diverse approaches: T4+TSH simultaneously, T4-reflex TSH and TSH-reflex T4	Diverse periods ranging from first week to first month of life	Diverse approaches	(9)
USA (Northwest Regional NBS Program)	T4-reflex TSH	First NBS 1-2 days of life, second NBS 10-14 days of life	Referral if T4 <10^th^ percentile	(4)
Israel (national program)	T4-reflex TSH	48-72 hours of life	Referral if T4 <10^th^ percentile	(10)
The Netherlands (national program)	T4-reflex TSH-reflex TBG	4-7 days of life	Referral if T4 ≤-3 SD and TBG >40 nmol/L; if T4 <-1.6 SD, then TBG measurement; referral if T4/TBG ratio ≤17 in 1^st^ and 2^nd^ NBS result	(11, 12)

Since the start of NBS, the incidence of central CH in the Netherlands has steadily increased from 1:106,304 in the late 1970s/early 1980s to approximately 1:16,404 in the last two decades (15, 16). Recently, van Iersel et al. showed that between 2001 and 2011 29 children with probable central CH as part of CPHD were missed by the Dutch NBS program over a 10-year period. This would raise the Dutch incidence from 1:16,404 to around 1:13,000, consistent with data from Japan (7, 17).

During the past years the assumption that central CH is usually a mild condition has been refuted. Zwaveling-Soonawala et al. found that, based on initial FT4 concentration, more than half of all newborns with central CH have moderate to severe disease (18). The same applies to the notion that most neonates with central CH as part of CPHD are diagnosed clinically shortly after birth. In a recent study on clinical characteristics of Dutch central CH patients Naafs et al. reported that most neonates with central CH as part of CPHD were only diagnosed after notification of an abnormal NBS result, even though many patients were hospitalized in the first weeks of life for feeding problems, hypoglycemia or (prolonged) jaundice (19). This review provides an update on the etiology and prognosis of central CH, and a practical approach to diagnosis and management of this intriguing condition.

## Physiology And Pathophysiology

### Normal Hypothalamus-Pituitary-Thyroid Function

The thyroid hormones T4 (the prohormone) and triiodothyronine (T3; the active hormone) are produced and secreted by the thyroid gland in a molar ratio of about 11 to 1 (20). In serum, T4 and T3 are mostly bound to transporting proteins TBG, albumin and transthyretin; only a small part circulates as unbound free T4 and T3 (FT4 and FT3, respectively) (21).

TH enters target tissue cells by crossing the cell membrane facilitated by specific TH transporters. The intracellular amount of T3 depends on circulating T3, but a number of target tissues regulate their own optimal T3 content by enzymatically activating T4 into T3 by deiodinase types 1 and 2 (DIO1 and 2), or by converting T4 into the inactive TH reverse T3 (rT3) by deiodinase type 3 (DIO3); DIO3 also inactivates T3 into T2 (20). T3 exerts its action by binding to the thyroid hormone receptor alpha or beta (TRα or TRβ).

TH production is regulated by the hypothalamus-pituitary-thyroid (HPT)-axis, a classic endocrine negative feedback system. Depending on the amount of circulating TH the hypothalamus and pituitary adjust thyroid function by secreting more or less thyrotropin releasing hormone (TRH) and TSH, respectively, and thereby to maintain a stable serum TH/FT4 concentration (22). In healthy individuals TSH and FT4 concentrations show small variability while interindividual variability is large, as judged from the wide laboratory reference intervals. This suggests that each individual has their own specific HPT-axis set-point.

### Central vs. Primary Hypothyroidism and Their Biochemical Characteristics

Hypothyroidism is defined as inadequate TH production causing TH deficiency at the target tissue level.

Primary hypothyroidism is caused by a defective thyroid gland and is characterized by a serum FT4 concentration below, and a TSH concentration above the reference interval. In subclinical (primary) hypothyroidism the serum FT4 concentration is still within the reference interval.

In central hypothyroidism the cause of the inadequate TH production is at the level of the hypothalamus or pituitary. In this scenario, decreased TH secretion is caused by quantitative or qualitative TSH deficiency (1). Biochemically, central hypothyroidism is defined as a FT4 concentration below the reference interval in combination with a normal, low, or slightly elevated TSH. The slightly elevated TSH concentrations observed in central hypothyroidism are partly explained by intact immunoactivity, but decreased bioactivity (23). Central hypothyroidism can be congenital or acquired (e.g., pituitary dysfunction after brain irradiation or trauma). While central CH may occur in isolation (TSH deficiency only), it is more often accompanied by other pituitary hormone deficiencies (combined pituitary hormone deficiency, CPHD (1).

In acquired central hypothyroidism, TH secretion can be reduced, while serum FT4 is still within the reference interval. This is explained by the abovementioned interindividual differences in HPT axis’ set-point. This state is referred to as subclinical central hypothyroidism (24).

While diagnosing primary hypothyroidism is based on the finding of a clearly elevated TSH concentration, diagnosing central hypothyroidism relies on correct interpretation of FT4 concentration using age-specific reference intervals to recognize when FT4 is too low. However, the individual set-point may complicate correct interpretation. For example, while an FT4 concentration in the lower range of the reference interval may be normal for most individuals it does not necessarily exclude central hypothyroidism, especially in individuals with a set-point in the higher range. Yet, in many instances central hypothyroidism may be a rather straightforward diagnosis, when serum FT4 is clearly below the reference range, signs or symptoms of hypothyroidism are present and the medical history is suggestive for hypothalamic or pituitary damage or disease. This is often the case in acquired forms of central hypothyroidism, but also in congenital central hypothyroidism as part of CPHD (1).

Diagnosis is further complicated when clear signs or symptoms are absent and the medical history is uninformative, as can be the case with newborns with an abnormal NBS result suggestive for central CH; then a FT4 concentration around the lower limit of the reference interval may pose a diagnostic dilemma (by definition, 2.5% of healthy individuals have an FT4 below the reference interval).

In recent years knowledge about the etiology of central CH has increased significantly. With a combination of diagnostics, like basal and dynamic endocrine investigation, genetic testing and high-resolution magnetic resonance imaging (MRI) an increasing percentage of previously uncertain diagnoses can be confirmed.

In the next section, the various causes of isolated central CH and CH as part of CPHD are discussed in detail.

## Etiology

### Isolated Central CH

Currently, five genes related to isolated central CH have been identified. Molecular analyses of Mendelian forms in the 1980s and 1990s allowed recognition of pathogenic variants of the *TSHB* and *TRHR* genes, associated with TSH deficiency. Implementation of next-generation sequencing-based genetic testing in cohorts of central CH patients has significantly increased our knowledge of the etiology and clinical spectrum of isolated central CH. Recently, three other genes were implicated in isolated central CH: *IGSF1*, *TBL1X* and *IRS4* (18, 19, 25, 26). These relatively common forms of isolated central CH are X-linked, so most patients are boys. Cases of females with a mild central hypothyroidism phenotype carrying a mono-allelic pathogenic variant of these X-linked genes have been described. Isolated central CH is a monogenic disorder in all known cases. In a recent study, a high percentage of cases of isolated central CH could be explained by pathogenic variants in the five aforementioned genes (19). However, a number of cases remained unexplained, suggesting currently unrecognized pathogenic variants in other genes or perhaps a polygenic etiology. In patients with isolated central CH, structural hypothalamic-pituitary (HP) abnormalities have not been found. Severe neonatal health problems are rare. Older, seemingly asymptomatic patients have been detected through familial segregation analyses of probands with isolated central CH, stressing the rather mild character of some subtypes.

#### TSHB

*TSHB* (OMIM #188540) was the first gene associated with isolated central CH. It encodes the beta subunit of TSH, which belongs to the family of glycoprotein hormones. The alpha subunit of TSH (encoded by *GCA*) is common to the pituitary and placental glycoprotein hormones TSH, luteinizing hormone and follicle stimulating hormone (LH&FSH) and human chorionic gonadotropin (hCG), while the TSH beta subunit grants biological specificity to TSH. The first patients with isolated central CH due to *TSHB* pathogenic variants carried the homozygous c.145G>A p.(Gly49Arg) variant (NM_000549.4) (27). This variant affects the evolutionarily conserved CAGYC region (residues 47-51), which is important for normal folding and TSH-α/TSH-β dimerization. The phenotype, consisting of quantitative TSH deficiency associated with mostly severe hypothyroidism, segregated in an autosomal recessive pattern. Later, patients were identified with different variants associated with qualitative TSH deficiencies characterized by measurable immunoreactive TSH without biological activity due to abolishment of signaling-specific domains but preserved TSH assay epitopes (28).

**Figure 1:**
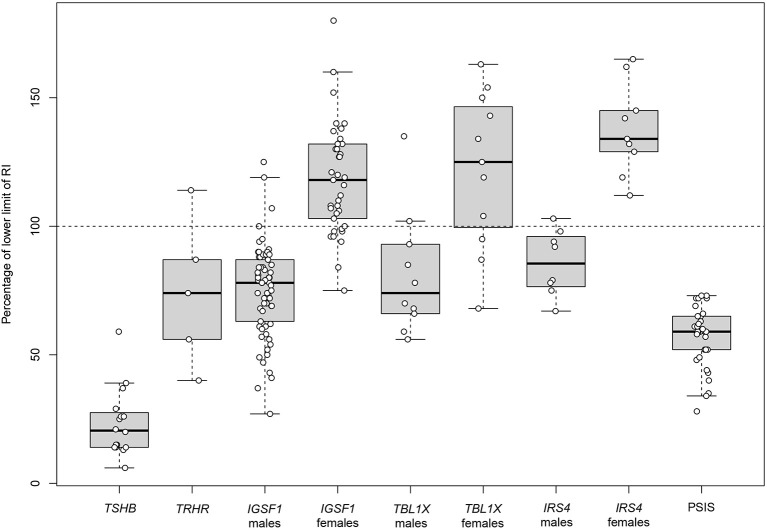
Pre-treatment serum FT4 concentrations in patients with central congenital hypothyroidism. First pre-treatment serum FT4 concentrations in patients with isolated central CH and in patients with central CH as part of CPHD caused by PSIS. Data of isolated central CH were extracted from published studies/case reports (**Supplementary Table 1**), and expressed as percentage of the lower limit of given FT4 reference intervals. Data about central CH as part of PSIS were extracted from the study by Naafs et al. (19). FT4 values of PSIS patients measured at day 3-7 and day 12-16 after birth were compared to the lower limit of the plasma FT4 reference interval at these ages (20.5 pmol/L and 15.3 pmol/L, respectively) (31). Boxes represent median, 1st and 3rd quartile, and whiskers represent minimum and maximum. Individual data points are given as circles. CH, congenital hypothyroidism; CPHD, combined pituitary hormone deficiencies; FT4, free thyroxine; PSIS, pituitary stalk interruption syndrome; RI, reference interval.

Given the substantial number of false-positives due to TBG deficiency in many T4-based NBS programs, it was stated that identification of heterozygous carriers of *TSHB* variants was preferable over T4-based NBS (29). Indeed, *TSHB* variants appear to be a very rare cause of isolated central CH (19), and the prevailing c.373del p.(Cys125Valfs*10) (NM_000549.4) variant was linked to a founder effect. However, the effectiveness of this approach has never been proven. Late diagnosed patients present with severe neurological deficits, while early treatment secures normal development (30). A meta-analysis of all recorded pre-treatment FT4 values underlines the severity of hypothyroidism due to biallelic *TSHB* variants (**Figure 1**).

#### TRHR

Not long after the discovery of pathogenic *TSHB* variants in isolated central CH, patients with biallelic pathogenic *TRHR* (OMIM #188545) variants were identified (32). *TRHR* encodes the TRH receptor, which is mainly expressed in thyrotrophs and lactotrophs. TRH is transported from the hypothalamus to the pituitary through the portal system to stimulate TSH and prolactin secretion. A logical assumption would be that beside *TSHB* and *TRHR* also the *TRH* (OMIM #613879) gene (encoding TRH) might be implicated in isolated central CH. Until now pathogenic variants of *TRH* have not been associated with central hypothyroidism in humans, but *Trh* knock-out mice have been generated (33).

Although a biallelic defective *TSHB* leads to severe hypothyroidism, this is not the case in defective *TRHR* (**Figure 1**). Different pathogenic variants underlie differences in TRH resistance (34, 35). Interestingly, even complete TRH resistance does not abolish TSHB and prolactin expression (35), an important insight into the molecular control of the HPT axis. The phenotype of moderate to mild central CH segregates in an autosomal recessive inheritance pattern, and may remain asymptomatic. However, treatment of asymptomatic patients is reported to improve their quality of life (QoL) (35). Heterozygous carriers of pathogenic *TRHR* variants with a mild effect on TRH resistance may have an elevated serum TSH; on the other hand, isolated TSH elevation instead of central hypothyroidism has been reported in homozygous carriers (34).

#### IGSF1

The first patients with a recessive X-linked isolated central CH phenotype were reported in 2012 (36). Pathogenic variants in the immunoglobulin superfamily member 1 gene (*IGSF1*, OMIM #300137) were identified in 11 families. Until now, more than 40 pathogenic *IGSF1* variants have been described, and it is the most prevalent cause of moderate to mild isolated central CH in males and females (19).

In addition to central CH, IGSF1 deficiency in males leads to a heterogeneous phenotype, including macroorchidism, delayed pubertal testosterone rise (but a normal timing of testicular growth) and in some cases other pituitary hormone deficiencies, namely low serum prolactin and growth hormone (GH) deficiency (in childhood) (**Table 2**) (37, 38). Peculiarly, adult males exhibit increased growth hormone secretion, and as a result acromegaloid features (39). While IGSF1 deficiency segregates in a recessive X-linked pattern, females carrying a pathogenic variant present with varying FT4 concentrations, ranging from moderately reduced to normal (**Figure 1**). Other characteristics include delayed menarche, low prolactin and increased BMI. X-inactivation was studied in heterozygous female carriers to determine the cause of the observed female phenotypic heterogeneity; however, no correlation was established (37).

**Table 2:** Summary of key findings in the five genetic causes of isolated central congenital hypothyroidism.

Gene	Selected key findings	TRH test results
*TSHB*	Males and females (biallelic pathogenic variants): mostly severe hypothyroidism, TSH deficiency (quantitative or qualitative)	Severely reduced/absent TSH response, normal prolactin (PRL) response
*TRHR*	Males and females (biallelic pathogenic variants): moderate to mild hypothyroidism, elevated TSH	Normal or absent TSH and PRL responses
	Males and females (carriers): recurrent TSH elevation	
*IGSF1*	Males (hemizygous pathogenic variant): moderate to mild hypothyroidism, macroorchidism, delayed pubertal testosterone rise, low prolactin, increased BMI and fat percentage, growth hormone deficiency (childhood), acromegaloid facies (adulthood)	Normal or reduced TSH response, normal or reduced/absent PRL response
	Females (heterozygous pathogenic variant): low-normal FT4 values to mild hypothyroidism, delayed menarche, low prolactin, increased BMI and fat percentage, acromegaloid facies (adulthood)	
*TBL1X*	Males (hemizygous pathogenic variant): moderate to mild hypothyroidism, hearing deficits	Normal TSH response, normal PRL response
	Females (heterozygous pathogenic variant): low-normal FT4 values to mild hypothyroidism	
*IRS4*	Males (hemizygous pathogenic variant): mild hypothyroidism	Reduced TSH response, normal or slightly reduced PRL response
	Females (heterozygous pathogenic variant): low-normal FT4 values	

IGSF1 is expressed at high levels in testes and the pituitary, specifically in thyrotrophs, somatotrophs and lactotrophs (36). Its precise function within the context of the pituitary hormone axes and testicular function is still unknown (40). *Igsf1* knock-out mice have central hypothyroidism, decreased pituitary TRH receptor 1 mRNA expression and impaired responsiveness to TRH compared to healthy littermates (36, 41). This supports a role for impaired TRH action in the pathogenesis of the central CH. While mouse models have successfully recapitulated the IGSF1 deficiency central hypothyroidism phenotype (36, 41), they lack the testicular enlargement phenotype (42). Although macroorchidism may results from IGSF1 loss-of-function in testes (43), other *Igsf1* knock-out animal models need to be explored in order to further elucidate its underlying pathophysiological mechanism.

#### TBL1X

More recently the fourth molecular cause of isolated central CH was discovered. The first patients with a pathogenic variant in transducing-beta-like 1 gene (*TBL1X*; OMIM #300196) were identified in 2016 (44). An X-linked recessive inheritance pattern associated with an incomplete penetrance was observed. The phenotype consists of moderate to mild central hypothyroidism in hemizygous males, and mild central hypothyroidism to euthyroidism in heterozygous females (**Figure 1**). Again, X-inactivation studies could not explain phenotypic heterogeneity in females (44).

*TBL1X* mRNA is expressed in multiple tissues, including the pituitary and hypothalamus (44), and the TBL1X protein is an essential subunit of the nuclear receptor corepressors (CoRs) silencing mediator of retinoid and thyroid hormone receptors (SMRT) and nuclear receptor co-repressor1 (NCoR1). CoRs associate with unliganded thyroid hormone receptors (TRs) in order to repress transcription of genes that are positively regulated by T3. T3 binding results in the release of the corepressors and recruitment of coactivators leading to increased gene transcription. However, genes involved in the negative feedback regulation of the HPT-axis like *TSHB* and *TRH* are negatively regulated by T3 which means that their transcription is increased in the absence of T3. It was shown that the c.1145_1147del p.(Asn382del), c.1246A>T p.(Asn416Tyr) and c.1526A>G p.(Tyr509Cys) (NM_005647.3) variants resulted in reduced stimulation of *TSHB* and *TRH* promoters in a hypothalamic cell line (45, 46). Consequently, this may alter HPT-axis functioning to the point that the hypothalamus and pituitary are resistant to low TH levels, with a possible negative shift of the FT4 set-point.

Furthermore, some patients exhibit hearing thresholds lower than age-specific reference intervals, unrelated to pre-treatment serum FT4 levels (44, 47). It was suggested that due to TBL1X and subsequently NCoR-SMRT dysfunction there is differential expression of T3-regulated genes involved in ear development, such as pendrin (47). Similarly, the homologous gene *TBL1Y* is implicated in X-linked hearing loss (48).

An important question concerning the severity of the central hypothyroidism phenotype remains. It is not precisely known what the effect of *TBL1X* variants is on target tissue TH action. In light of this issue, patients with pathogenic *TBL1X* variants have normal TSH and FT4 responses to exogenous TRH administration, hinting at functional integrity of the pituitary. Also, patients that were identified at an older age have presented in seemingly asymptomatic states with normal intellectual development (44). A mouse model with a disturbed liver specific NCoR–TR binding showed upregulation of positive TH target genes in the presence of T3 (49). Accordingly, mice with global expression of this NCoR variant had biochemical features associated with central hypothyroidism, but normal growth and increased energy expenditure (50). Low TH and normal TSH concentrations in these mice may be an adaptive HPT-axis response to increased target tissue TH sensitivity. Deleterious *TBL1X* variants could have the same effect on transcription of positive TH target genes, but this has yet to be proven in animal studies.

**Figure 2:**
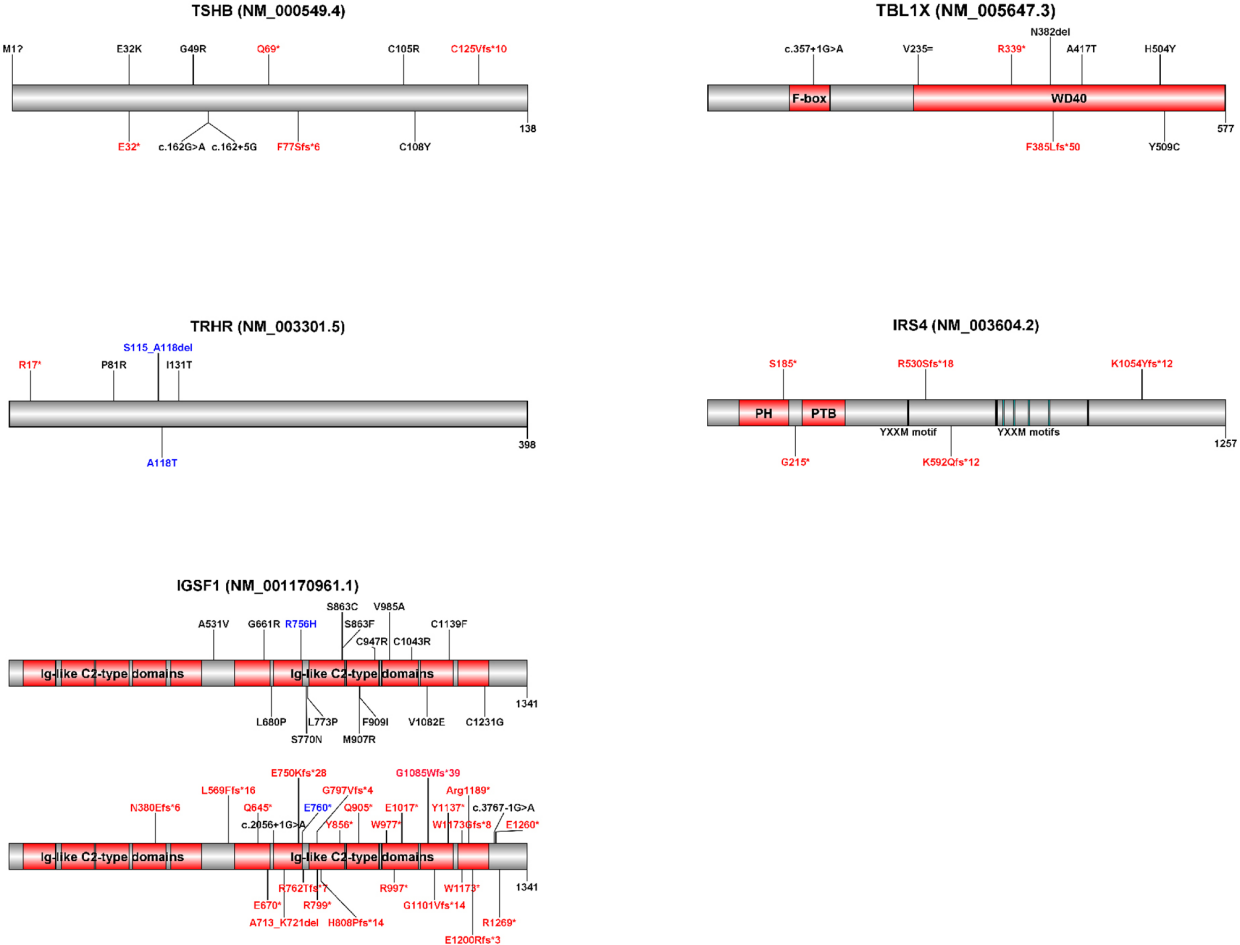
Schematic representations of the five genes implicated in isolated central CH with known pathogenic variants. Variants displayed in red represent premature termination codon variants. Variants displayed in black represent missense, synonymous and in-frame indel variants. Variants displayed in blue were found on the same allele. Variant annotations may differ from published variants due to differences between transcripts (indicated in brackets). Diagrams were created with Domain Graph, version 2.0 (http://dog.biocuckoo.org/). Ig, immunoglobulin; PH, pleckstrin homology domain; PTB, phosphotyrosine binding domain.

Reported *TBL1X* variants mainly involve the WD40 domain (**Figure 2**), necessary for protein-protein and protein-chromatin interactions in the context of NCoR-SMRT action (44, 46, 47). In the first report on loss-of-function variants in *TBL1X*, families were identified with missense variants in the WD40 domain (44). Later, the phenotype of a boy with a *de novo* nonsense variant in the WD40 domain was described (47). The boy had central hypothyroidism and sensorineural hearing deficits like the previously identified patients, but also other signs that could be linked to NCoR-SMRT dysfunction, including attention deficit/hyperactivity disorder (ADHD), learning difficulties, fecal incontinence and Chiari malformation type I. More patients with premature termination codon variants need to be identified before its relation to these symptoms can be established. A phenotypic spectrum of TBL1X deficiency due to variable degrees of NCoR-SMRT dysfunction seems plausible.

#### IRS4

The most recently discovered genetic cause of isolated central CH was reported in 2018 (51). Five male probands from unrelated families with pathogenic variants in the insulin receptor substrate-4 gene (*IRS4*) (OMIM #300904) had a phenotype consisting of solely X-linked central hypothyroidism and an abnormal TSH response to exogenous TRH. One patient had mild TSH elevation aside from low FT4. Females carrying a heterozygous pathogenic variant had low-normal to normal FT4 concentrations (**Figure 1**). All reported variants are premature termination codon variants. No other reports on the further phenotypic delineation have emerged since the first report, however additional patients have been identified (19).

*IRS4* is expressed in hypothalamic nuclei, pituitary gland, ovaries, uterus, the thyroid gland and digestive tract among others (51, 52). *IRS4* encodes for the adaptor protein IRS4, which belongs to the family of IRS proteins. *IRS1* and *IRS2* have a widely distributed expression but limited shared amino acid sequence with *IRS4* (53). *IRS3* is a pseudogene in humans, and *IRS5* and *IRS6* have a more specific expression pattern, in liver and kidney, and skeletal muscle, respectively. Based on phylogeny studies, it was postulated that the amino acid sequence of IRS4 accelerated in early mammalian development, thus having distinct, but also overlapping functions compared with the other IRS proteins (53). IRS4 functions as an interface between hormone receptors with tyrosine or serine/threonine kinase activity (for example the insulin receptor, IGF1 receptor, FGF receptor 1, and BMP receptor 2), and signaling molecules with an SH2 domain (54, 55). Upon ligand activation of tyrosine or serine/threonine kinase receptors, intracellular sites are phosphorylated. IRS4 interacts with these phosphorylated sites, which results in phosphorylation of tyrosine or serine/threonine phosphorylation motifs. SH2 domain-containing proteins are then recruited and activated (54). Downstream signaling pathways of IRS4 include the MAPK cascade and the PI3K/AKT/mTOR pathway (56).

The pathophysiological mechanism of IRS4 pathogenic variants has not been elucidated yet. Several mouse models have failed to recapitulate the phenotype (51, 57, 58), possibly due to redundancy and overlapping functions of IRS proteins (53, 58–60). It was suggested that the central hypothyroidism phenotype is linked to leptin receptor (LEPR) dysfunction. Fasting signals are conveyed to hypothalamus through leptin/LEPR signaling, modulating *TRH* expression (61, 62). Since IRS4 is implicated in leptin/LEPR signaling (63), *IRS4* loss-of-function hypothetically diminishes *TRH* expression. Indeed, fasted subjects (an example of subjects with reduced leptin signaling) have similar TSH secretion patterns and TRH test results as patients with central hypothyroidism due to *IRS4* loss-of-function (51). However, patients with leptin deficiency and leptin resistance invariably present with central hypothyroidism, and have a different TSH secretion pattern in response to exogenous TRH than patients with *IRS4* loss-of-function. Moreover, patients with IRS4 dysfunction have blunted TSH responses to exogenous TRH, hinting at a defect at the level of the pituitary. The precise functions of IRS4 in the pituitary remain unclear. A pathophysiological mechanism of central hypothyroidism due to IRS4 dysfunction incorporating pituitary TRH resistance or defective TSH transcription/post-translational modification is conceivable.

**Table 2** shows the key findings in the five genetic causes of isolated central CH. **Figure 2** shows schematic representations of the five genes implicated in isolated central CH with known pathogenic variants. **Supplementary Table 1** shows all reported pathogenic isolated central CH variants.

### Central CH as Part of CPHD

Besides occurring in isolation central CH may be part of CPHD. During embryonic brain development complex cascades of signaling molecules and transcription factors influence midline brain structure formation, including HP development. The anterior pituitary lobe derives from oral ectoderm and consists of five specialized cell types, secreting six hormones: thyrotrophs producing TSH, somatotrophs producing GH, corticotrophs producing adrenocorticotropic hormone (ACTH), gonadotrophs producing LH and FSH, and lactotrophs producing prolactin. The posterior pituitary lobe derives from neural ectoderm and stores and secretes the hypothalamic produced hormones vasopressin and oxytocin. The anterior pituitary cell types receive input from hypothalamic hormones *via* the hypophyseal portal blood vessels in the pituitary stalk. The posterior pituitary has a neural connection with the hypothalamus by way of the hypothalamic-neurohypophyseal tract in the pituitary stalk (64).

CPHD is characterized by a very broad genetic and phenotypic heterogeneity with variable penetrance. In addition, CPHD not only seems to be of monogenic but also of di- and oligogenic origin. Defects in one or more genes involved in midline brain formation result in a variety of malformations. Genetic defects in factors involved in early embryonic brain development result in the most severe midline brain anomalies, while defects in transcription factors involved in the final steps of pituitary cellular differentiation cause milder CPHD without structural pituitary malformation (*POU1F1*, *PROP1*).

We divide CPHD into three main categories: 1) CPHD without pituitary malformation, 2) CPHD with isolated pituitary malformation, and 3) syndromic CPHD with pituitary and extra-pituitary malformations. The combination of pituitary hormone deficiencies and the severity are very variable. Overall GH deficiency is the most common endocrine deficit in CPHD, followed by central CH.

#### CPHD Without Pituitary Malformation

##### POU1F1

*POU1F1* (previously known as *PIT1*) (OMIM #173110) was the first gene to be identified in CPHD patients. It is a pituitary-specific transcription factor expressed in the anterior pituitary, relatively late during pituitary development. It is responsible for differentiation of somatotroph, lactotroph and thyrotroph cells. In 1992 the first homozygous nonsense variant of *POU1F1* was identified in a CPHD patient from consanguineous parents (65).

Since this first description 38 pathogenic variants have been reported. Most are associated with an autosomal recessive inheritance pattern although a few autosomal dominant variants have been described. CPHD consists of TSH, GH and prolactin deficiency with normal gonadotroph and corticotroph axes. Patients often present at a very young age with growth retardation due to severe GH deficiency. Presentation of central CH is variable. Most patients have TSH deficiency early on, but it may also occur later in childhood (66). Also cases with normal TSH have been reported. Neuroimaging usually shows a normal or hypoplastic anterior pituitary lobe, with a normal posterior lobe and pituitary stalk. *POU1F1* pathogenic variants are found in approximately 2.6% of non-familial CPHD cases, and in up to 21.6% in familial CPHD cases (67).

##### PROP1

In 1998 the first four families with CPHD due to homozygous and compound heterozygous *PROP1* (OMIM #601538) variants were described. Today, *PROP1* is the most frequent genetic cause of CPHD with a eutopic posterior pituitary (68). PROP1 is a transcription factor involved in differentiation of somatotroph, lactotroph, gonadotroph and thyrotroph lineages. CPHD consist of GH, prolactin and TSH deficiency, in addition to variable deficiency in LH&FSH and ACTH. Most affected individuals present with growth failure in infancy or early childhood due to severe GH deficiency. Central CH is usually mild and present later in infancy or childhood. ACTH deficiency occurs later on with a reported mean age at diagnosis of 25.3 years (range 7.4-67 years) (68). Most patients have a small or normal anterior pituitary with a normal pituitary stalk and posterior pituitary. Some cases of enlarged anterior pituitary gland during childhood have been described with subsequent involution over time. *PROP1* pathogenic variants are found in approximately 11% of CPHD patients with a frequency of up to 50% in familial cases and 7% in sporadic cases.

#### Isolated Pituitary Stalk Interruption Syndrome

**Figure 3:**
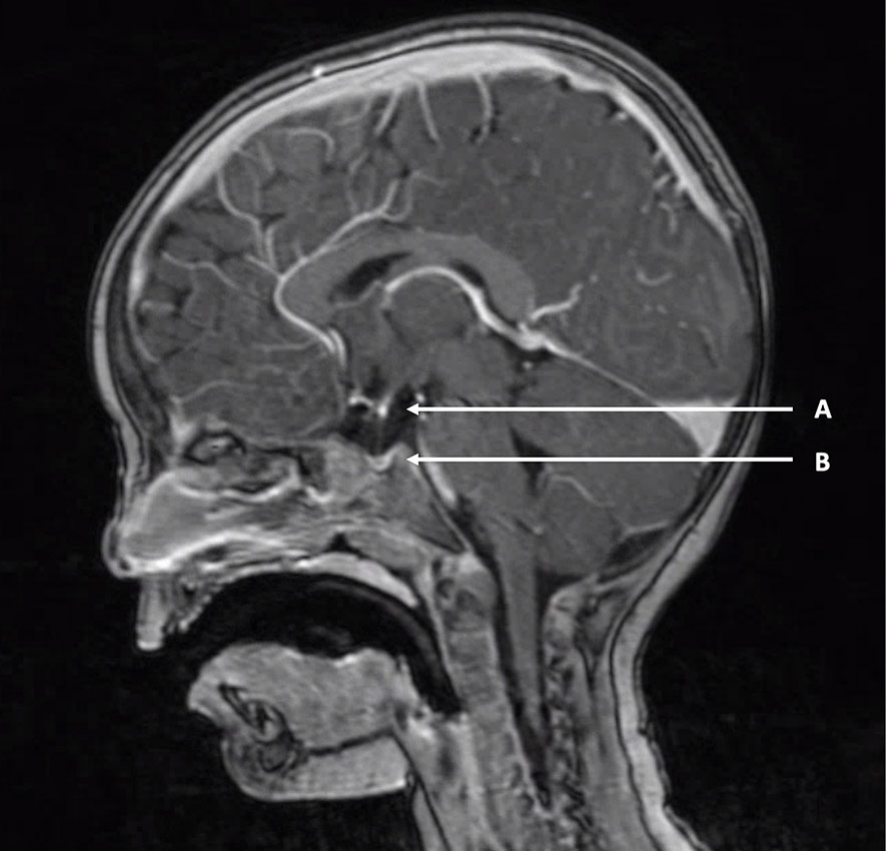
Example of pituitary stalk interruption syndrome. T1-weighted MRI of a two-year-old boy with pituitary stalk interruption syndrome. **(A)** “Bright white spot” of ectopic posterior pituitary positioned at the hypothalamic base. **(B)** Hypoplastic anterior pituitary in the sella turcica. A thin pituitary stalk is visible between **(A, B)**.

The most common isolated pituitary malformation is referred to as pituitary stalk interruption syndrome (PSIS), and consists of a classic triad of interrupted or absent pituitary stalk, ectopic posterior pituitary and anterior pituitary hypoplasia or aplasia (**Figure 3**). It was first described in 1987 in a series of ten patients with “idiopathic pituitary dwarfism” showing an ectopic pituitary lobe and transection of the pituitary stalk upon magnetic resonance imaging (MRI) (69). Since then, there have been many reports on PSIS and a broad clinical, radiological and genetic heterogeneity has become apparent (70–72). The classic triad may not always be complete, but cardinal features include either interrupted/absent pituitary stalk or ectopic posterior pituitary. The position of the posterior pituitary varies and may be at the hypothalamic base along the pituitary stalk, or even in the normal position within the sella turcica. PSIS is considered a midline brain malformation and regarded to be at the mild end of the holoprosencephaly spectrum. PSIS most often occurs in isolation but it may also be associated with extra-pituitary malformations, mainly affecting brain, eye and craniofacial structures. The clinical phenotype of PSIS consists of anterior pituitary hormone deficiencies in variable combinations with normal posterior pituitary function. The normal posterior pituitary function reflects an intact neural hypothalamic-posterior pituitary connection while the hypothalamic-anterior pituitary connection, through the pituitary stalk, is disrupted.

Patients with PSIS may present immediately after birth with classic signs of hypopituitarism such as hypoglycemia, prolonged jaundice, undescended testes/micro-penis. Most often, however, patients present later on in childhood with poor growth. In a twenty-year cohort of 154 Dutch central CH patients detected by neonatal screening 39% (60/154) had isolated central CH and 61% (94/154) had CPHD. Of these 94 CPHD patients, 85% (80/94) had isolated PSIS and 15% (14/154) had syndromic PSIS (19). This makes isolated PSIS the most frequent diagnosis in central CH patients detected by the Dutch neonatal screening. In addition to central CH, 96% of the patients had GH deficiency, 86% ACTH deficiency and 74% LH&FSH deficiency. Posterior pituitary function was normal in all patients. The Dutch cohort consists of a specific subset of PSIS patients who all have central CH and therefore does not represent the whole spectrum of PSIS. Bar et al. reported on clinically detected PSIS patients including ten patients with a neonatal diagnosis and 47 patients diagnosed after presentation for growth retardation at a mean age of four years. Central CH was present in 80% of these patients (72). PSIS is also encountered in patients with isolated GH deficiency and this may be regarded the mildest phenotype of PSIS. However, in patients with an initial diagnosis of isolated GH deficiency, GH treatment frequently leads to a marked decrease in serum FT4 and thereby unmasks the diagnosis of central CH. In these patients with unmasked central CH after GH treatment, MRI usually reveals PSIS (17). Pituitary hormone deficiencies in PSIS are not always present at birth and may evolve throughout life, which is especially the case for ACTH deficiency. Therefore, long-term monitoring for development of additional anterior hormone deficiencies advised. In line with this all patients with apparently isolated GH deficiency should have close monitoring of FT4 levels under treatment and undergo pituitary MRI (73). See also the section on treatment and follow-up.

Compared to isolated forms of central CH due to *IGSF1*, *TBL1X* and *IRS4* variants, FT4 concentrations in PSIS patients are usually lower and classify as moderate severe hypothyroidism (18). TSH concentrations in central CH due to PSIS are usually normal to slightly elevated. In the Dutch cohort of isolated PSIS patients the highest TSH measured was 12.9 mIUl/L (74). Mildly elevated TSH in PSIS is probably due to the absence of normal TRH stimulation leading to impaired TSH glycosylation and resulting in decreased bioactivity of circulating TSH. Since TSH values in isolated central CH cases are reported to be in the low-normal range, a mildly raised TSH concentration is suggestive of PSIS. Although the value of TRH-testing in the diagnosis of central CH is debated, in the Dutch cohort almost all PSIS patients had a delayed TSH rise (19). Ultimately MRI is the key to a definitive PSIS diagnosis. The posterior pituitary has a marked hyperintensity upon T1-weighted images making it recognizable as the “bright white spot” (**Figure 3**). While an ectopic position of the bright white spot is indicative of PSIS, absence of the bright white spot is found in up to 10 per cent of healthy individuals. In addition, the posterior pituitary hyperintensity may not be visible before the age of 2 months (75).

Monogenic causes of isolated PSIS are extremely rare and it has been suggested that, due to the low yield, genetic testing in non-familiar isolated PSIS cases is not indicated. Recent studies using next generation sequencing techniques have identified a few monogenic causes of isolated PSIS involving *CDON* (OMIM #608707), *GPR161* (OMIM #612250) and *GLI2* (OMIM #165230) genes (76–78). These genes are all involved in the Sonic Hedgehog signaling (SHH) pathway emphasizing the important role of this pathway in midline brain development. Rather than a monogenic etiology, there is growing evidence for a digenic or polygenic etiology for isolated PSIS (76, 79, 80). PSIS seems to result from a combination of defects in various genes in the Wnt, Notch and Sonic Hedgehog pathways involved in midline brain development (71, 81).

#### Syndromic CPHD

In syndromic forms of CPHD, pituitary insufficiency is accompanied by other cerebral and extra-cerebral abnormalities. Cerebral structures often include midline brain, eye, inner ear and craniofacial structures. Midline brain malformations may consist of holoprosencephaly, septo-optic dysplasia (SOD), absent corpus callosum, cerebellar malformations, Arnold Chiari malformation and also PSIS (syndromic PSIS). Cleft lip or palate and dental malformations such as single central incisor are examples of craniofacial malformations. Extra-cerebral structures that may be involved are heart, urinary tract, gastro-intestinal tract and axial skeleton.

Obvious birth defects, neurological and developmental problems will usually lead to an early diagnosis of syndromic CPHD. The yield of genetic testing is low with a genetic abnormality found in only 5-10% of sporadic cases. With an incidence of 1 in 10,000 live births SOD is one of the more common syndromic forms of CPHD consisting of two of the three features: optic nerve hypoplasia, midline forebrain abnormalities and pituitary hypoplasia. Hypopituitarism is reported in around two thirds of SOD patients with variable severity. *HESX1* (OMIM #601802), *SOX2* (OMIM #184429), *SOX3* (OMIM #313430) and *OTX2* (OMIM #600037) gene defects have been implicated in the etiology of SOD, although genetic defects are only found in up to 10% of SOD cases (64). Although SOD is one of the more common syndromic forms of CPHD, the frequency of central CH among patients with this condition is not known. It has been reported that approximately 62% to 80% of patients with SOD have hypopituitarism, with GH deficiency being the most common abnormality, but data on central CH are lacking (82, 83).

## Diagnosis

### How Do Patients With Central CH Present?

As mentioned earlier, the incidence of central CH has increased about six-to eightfold in the past few decades. This probably reflects improved detection by NBS early in life and careful registration of affected children, rather than a true increase in incidence over time (2).

In countries that do not screen for central CH, affected children may be diagnosed clinically in the first weeks to months of life because of signs and symptoms of TH deficiency (see paragraph 4.2.2). When not clinically diagnosed early in life, these children may present later on with developmental delay (resulting from TH deficiency ± neonatal hypoglycemia), poor growth (GH deficiency) and delayed puberty (LH&FSH deficiency).

Since serum T4 concentrations in premature born infants are low these children are not screened for central CH, as this would result in a large number of false positive results. In the Dutch NBS program, for premature born children (gestational age ≤36 weeks and birth weight ≤2500 grams) only the TSH concentration is taken into account. Thereby primary CH is detected, but central CH is missed (11, 16).

In countries, states or regions with NBS for central CH many affected newborns are asymptomatic or only mildly symptomatic at presentation. Most children with central CH detected by NBS in the Netherlands have CPHD (around 60%) (19).Therefore, the benefit of early detection, diagnosis and treatment of central CH is prevention of possible neurological sequelae of TH deficiency or hypoglycemia due to GH deficiency and ACTH deficiency.

In order to allow early treatment of affected newborns and to avoid unnecessary treatment of false positives, *appropriate* and *rapid* diagnostics should be offered to all referred newborns.

### Diagnostics in Case of Suspected Central CH

**Figure 4:**
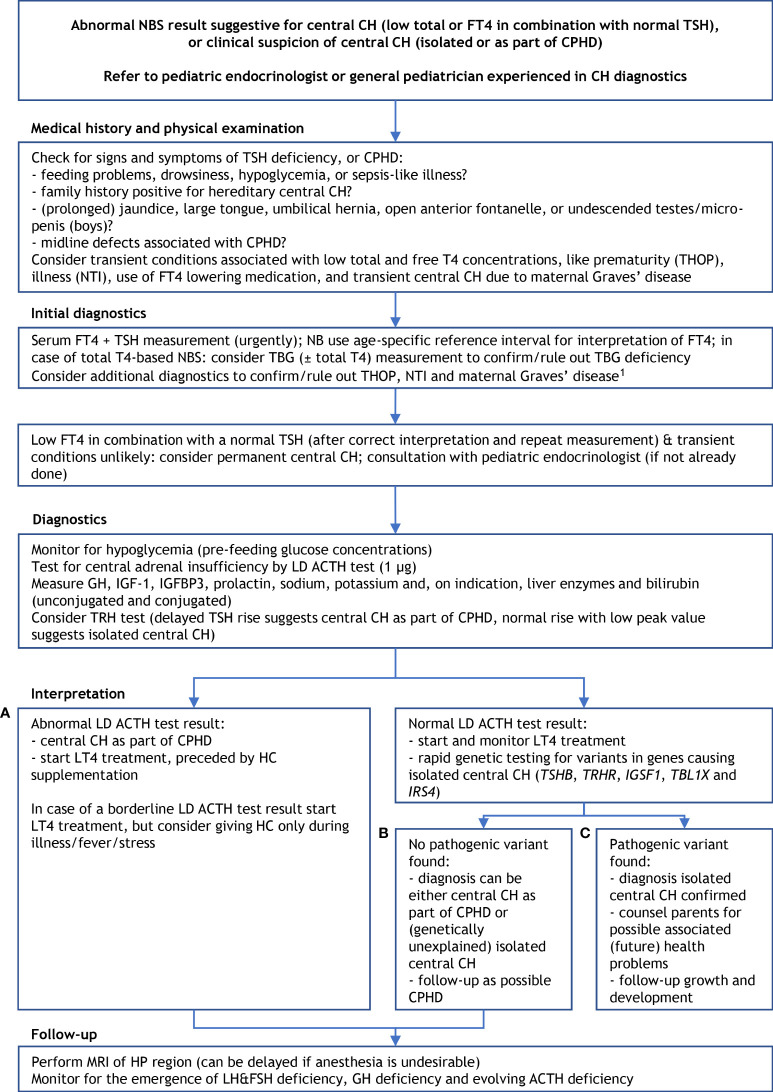
Proposed diagnostics after an abnormal newborn screening result suggestive for central congenital hypothyroidism. ^1^ suspicion of THOP: re-measure FT4 and TSH at term age, suspicion of NTI: measure (F)T3 ± rT3, re-measure FT4 and TSH at recovery; suspicion of transient central CH due to maternal Graves’ disease: measure TSHRAb and maternal thyroid function, and start thyroxine treatment when the newborn’s FT4 is too low. ACTH, adrenocorticotropic hormone; CH, congenital hypothyroidism; CPHD, combined pituitary hormone deficiency; FT4, free thyroxine; FSH, follicle stimulating hormone; GH, growth hormone; HC, hydrocortisone; HP, hypothalamic-pituitary; IGF-1, insulin-like growth factor 1; IGFBP3, insulin-like growth factor binding protein 3; LD, low dose; LH, luteinizing hormone; LT4, levothyroxine; MRI, magnetic resonance imaging; NBS, newborn screening; NTI, non-thyroidal illness; rT3, reverse triiodothyronine; T4, thyroxine; TBG, thyroxine binding globulin; TH, thyroid hormone; THOP, transient hypothyroxinemia of prematurity; TRH, thyrotropin releasing hormone; TSH, thyroid stimulating hormone; TSHRAb, TSH receptor antibodies. **(A–C)** refer to three scenarios of different outcomes of the work-up of central CH (see the *Management* section).

**Figure 4** shows a proposed diagnostic algorithm to be used in newborns/neonates suspected of central CH. This algorithm is used in the Netherlands, and although it is designed for use after an abnormal NBS result suggestive for central CH – low total or free T4 in combination with normal TSH –, the algorithm can also be used when central CH (isolated or as part of CPHD) is suspected on clinical grounds early in life.

#### Medical History and Physical Examination

The medical history and physical examination should focus on signs and symptoms of TH deficiency, and deficiencies of pituitary hormones other than TSH. In the first weeks to months of life TH deficiency may cause feeding problems, drowsiness, (prolonged) jaundice, large tongue, umbilical hernia, and open large anterior fontanelle. Central CH as part of CPHD may also present with hypoglycemia (due to ACTH ± GH deficiency), sepsis-like illness (ACTH deficiency), undescended testes/micro-penis (boys; LH&FSH deficiency), elevated liver enzymes (ACTH deficiency and TSH deficiency), and midline defects associated with CPHD.

In addition, attention should be paid to the presence of conditions associated with transient low total and free T4 concentrations, like transient hypothyroxinemia of prematurity (THOP), non-thyroidal illness (NTI; also known as sick euthyroid syndrome), use of FT4 lowering medication and transient central CH due to maternal Graves’ disease. The latter condition may be a reason for temporary levothyroxine (LT4) treatment (84–86).

#### Initial Diagnostics

In case of an abnormal NBS result, the initial step is confirmation of a low serum FT4 in combination with normal a TSH by (venous) blood collection. Optionally, TBG can be measured to test for TBG deficiency as cause of low NBS total T4 (2, 11). In the first two to three weeks of the neonatal period the lower limit of the FT4 reference interval lies considerably higher than later on; the same applies to the upper limit of the TSH reference interval (31, 87). This should be kept in mind when interpreting the measured FT4 and TSH concentrations. It may be necessary to perform additional diagnostics to confirm/rule out THOP, NTI and maternal Graves’ disease (**Figure 4**).

#### Diagnostics and Interpretation

Signs and symptoms of TH deficiency or of pituitary hormone deficiencies other than TSH are highly suggestive of the diagnoses isolated central CH or central CH as part of CPHD. The same applies to persistently low serum FT4 concentrations with normal TSH in the absence of aforementioned transient conditions. Additional support for the diagnosis central CH can be obtained by diagnosing one or more other pituitary deficiencies, finding variants in genes causing isolated central CH, or finding structural HP abnormalities associated with CPHD, like PSIS.

Management of children diagnosed with CPHD differs significantly from those with isolated central CH. Not only with respect to additional risks, but also regarding supplementary diagnostics and treatment. For instance, ACTH ± GH deficiency may cause hypoglycemia and warrant glucose monitoring. In case of ACTH deficiency, it is important to first start hydrocortisone treatment should be started before LT4, since LT4 treatment may provoke a potentially life-threatening adrenal crisis in patients with untreated ACTH deficiency. It is therefore important to test for ACTH deficiency early on (1, 88).

In our experience, this can be done by monitoring for hypoglycemia and by performing a low dose (LD, 1 µg) ACTH test (89). This may require a short hospital admission. If supplemented by (rapid) genetic testing for gene variants causing isolated central CH (which can be omitted if there is convincing evidence of additional pituitary hormone deficiencies, for instance a clearly abnormal LD ACTH test [that was technically well-executed]), there are three possible outcomes/scenarios:

A) abnormal LD ACTH test result;
B) normal LD ACTH test result but no pathogenic gene variant found;
C) normal LD ACTH test result and pathogenic variant in *TSHB*, *TRHR*, *IGSF1*, *TBL1X* or *IRS4*.

## Management

An abnormal LD ACTH test (scenario A) points to central CH as part of CPHD. In that case LT4 treatment should be preceded by hydrocortisone supplementation. In case of a borderline LD ACTH test result, consider prescribing hydrocortisone only during illness, or other forms of severe stress. LD ACTH testing is an error-prone procedure; requiring dilution of synthetic ACTH and my lead to a false abnormal test result. When doubting the reliability of the LD ACTH test result, consider rapid genetic testing for causes of isolated central CH. The finding of a pathogenic variant in one of the five isolated central CH genes will prevent unnecessary hydrocortisone treatment and additional diagnostics.

When the LD ACTH test result is normal, LT4 treatment can be started safely. Finding a pathogenic variant in *TSHB*, *TRHR*, *IGSF1*, *TBL1X* or *IRS4* (scenario C), confirms the diagnosis isolated central CH. Parents can be counseled on possible associated (future) health problems, like a small chance of partial GH deficiency and later on GH excess, delayed puberty and macroorchidism in IGSF1 deficiency syndrome, or hearing loss due to *TBL1X* loss-of-function (37, 44).

In scenario B – a normal LD ACTH test, but no pathogenic variant found -, the diagnosis can be either central CH as part of CPHD or (genetically unexplained) isolated central CH. In this scenario LT4 treatment can be started but as long as CPHD is not ruled out children should be followed-up as possible CPHD (scenario A and B). The diagnosis CPHD can be (further) substantiated by performing an MRI of the HP region and finding PSIS. MRI can be delayed if anesthesia is undesirable. A TRH test may be helpful in differentiating between central CH as part of CPHD and isolated central CH (90). A delayed TSH rise suggests central CH as part of CPHD, a normal rise but low peak value suggests isolated central CH (19, 90). A TRH test, however, should be performed before the start of LT4 treatment. Since ACTH deficiency may develop later on the importance of periodic testing for central adrenal insufficiency cannot be stressed enough.

In accordance with the recently updated CH consensus guidelines, treatment of central CH consists of once daily administration of LT4 (orally; tablets or liquid preparation) (91). In severe central CH (serum FT4 before treatment <5 pmol/L), the LT4 starting dose should be at least 10 μg/kg per day, in order to rapidly bring FT4 within the age-specific reference interval. To reduce the risk of overtreatment, a lower starting dose (5-10 μg/kg per day) can be used in milder forms. The (biochemical) long-term aim of LT4 treatment is to bring and keep serum FT4 in the *upper half* of the age-specific reference interval. Although randomized clinical trials testing this approach in children are lacking, data from adult studies support this approach (92, 93). The biggest difference between treatment of primary and central CH is in treatment monitoring, with serum FT4 (instead of TSH) being the most important parameter. If TSH prior to treatment was low, subsequent TSH measurements can be omitted. With regard to treatment monitoring, clinical and biochemical follow-up is similar to primary CH. The first evaluation should be scheduled one to two weeks after the start of treatment. Subsequent evaluations should take place every two weeks until normalization of serum FT4. Thereafter, the frequency can be lowered to once every one to three months until the age of 12 months, every two to four months during the second and third years of life, and every three to six months thereafter. Blood collection for serum FT4 measurement should be performed before, or at least four hours after the last (daily) LT4 administration (91). When under- or overtreatment is suspected, measurement of TSH, and free or total T3 may be helpful. Undertreatment should be considered when FT4 is around the lower limit of the reference interval, particularly if TSH >1.0 mIU/L. Overtreatment should be considered when FT4 is around or above the upper limit of the reference interval, and accompanied by clinical signs of thyrotoxicosis or a high (F)T3 concentration (1).

As already mentioned, in a study on the clinical characteristics of 92 Dutch children with central CH early detected by neonatal screening (57 as part of CPHD, 35 isolated disease), Naafs et al. found that 86% of the patients with CPHD also had ACTH deficiency, and that 96% and 74% had GH and LH&FSH deficiency, respectively (19). Most cases of ACTH deficiency (35 of 49) were diagnosed in the neonatal period, but nine cases between the ages of two months and one year, and the remaining five cases between the ages of one and 14 years. GH deficiency was diagnosed at a mean age of 1.3 years (range age 21 days to 9.2 years), and LH&FSH deficiency both in the first months of life and around the age of 12.8 years.

The implications of these findings are that all children diagnosed with central CH as part of CPHD, and all children without a genetically confirmed diagnosis of isolated central CH should be followed-up and monitored for the emergence of LH&FSH deficiency, GH deficiency and, evolving ACTH deficiency.

## Prognosis

Since the start of NBS for CH the neurodevelopmental prognosis of early detected and treated children with primary CH has improved dramatically (91). In 1980, Klein reported a mean IQ of 76 observed in over 800 patients with primary CH clinically diagnosed before the era of NBS (94). More recently, in a systematic literature search and review, Grosse et al. identified four population-based studies conducted in high income countries and found a somewhat higher mean IQ of 85 among children with clinically diagnosed primary CH prior to the introduction of NBS; 8-28% of these children were classified as having intellectual disability (defined as an IQ <70) (95).

In contrast, the latest long-term follow-up studies in patients with primary CH show that when LT4 treatment is started before the (mean) age of 10 days with a starting dose of at least 10 μg/kg per day, affected children have a neurodevelopmental outcome similar to that of unaffected siblings in the second decade of life (91, 96, 97). Although randomized clinical trials have not been conducted, the results of these studies indicate that early detection and adequate LT4 treatment of newborns with primary CH prevents brain damage and developmental delay.

Comparable outcome data of children with central CH are scarce. In a recent systematic review with meta-analysis of individual patient data, Naafs et al. identified six studies of reasonable quality on neurodevelopmental outcome in patients treated for central CH from which data of only 30 patients (27 with central CH as part of CPHD) was sufficient for analysis. While mean full scale intelligence quotient (FSIQ) was normal in these 30 patients (97; 95% confidence interval (CI) 88-105), 27% had a FSIQ below 85 (≥1 SD below norm score), and 10% below 70 (≥2 SD below norm score). Since in half of the studies the age at start of treatment was not available reliable conclusions could not be drawn (98). Subsequently, Naafs et al. performed a study on cognitive and motor outcome in a large cohort of Dutch patients with early-detected central CH (26). In this cross-sectional study, FSIQ was measured in 52 patients with CPHD and 35 patients with isolated central CH born between 1995 and 2015, with 52 unaffected siblings as controls. Secondary outcomes were intelligence tests’ subscales and motor function. Mean FSIQ was 90.7 (95% CI 86.4-95.0) in CPHD patients and 98.2 (95% CI 93.0-103.5) in isolated central CH patients. CPHD patients had lower FSIQs than siblings (mean difference -7.9 points, 95% CI -13.4 to -2.5), but FSIQs of isolated central CH patients and siblings were similar. Processing speed was lower in both patient groups than in siblings; motor difficulties occurred significantly more often in patients (33%) versus siblings (5%; p=0.004).

These data suggest that early detection and TH treatment of newborns with isolated central CH results in a normal IQ later in life, like in patients with early detected and treated primary CH. The approximately 0.8 SD lower IQ in patients with CPHD may be explained by ACTH and GH deficiency (present in 88% and 96% of patients, respectively), resulting in neonatal hypoglycemia (documented in 55% of patients; mean lowest glucose level 1.2 ± 0.8 mmol/L). Just like in primary CH, the lower processing speed and the more frequent occurrence of motor difficulties in isolated central CH and in CPDH patients may be related to pre- and early postnatal TH deficiency (26).

Naafs et al. also studied health-related QoL in the same cohort of patients with early-detected central CH and their unaffected siblings (99). Patients ≥8 years old filled in self-reports, and parents of patients aged three to 18 years old filled in parent-reports of the Pediatric Quality of Life inventory (PedsQL™) and the PedsQL Multidimensional Fatigue Scale. Patients with isolated central CH (n=35) and siblings showed similar scores on all subscales, both in the self-reports and parent-reports. Self-reported sores of CPHD patients (n=53) were also similar to those of siblings. However, parent-reported total HRQoL and fatigue scores of CPHD patients were significantly lower than those of siblings. This indicates a perceived difference in perception between patients and their parents.

Despite these positive and promising results, the remaining question is whether early detection and treatment of children with central CH really improves their neurodevelopmental outcome and, if so, how much. Although a few studies not included in the systematic review by Naafs et al. suggest that it does (100, 101), this question can only be answered by conducting neurodevelopmental outcome studies in patients with late-detected central CH. Since isolated CH may not be clinically detected at all, such studies may have to focus on patients with CPHD due to PSIS, which was present in 88% of the patients evaluated by Naafs et al. (19) Given the rarity of this disorder this can only be achieved by international collaboration for instance in the form of an international patient registry.

## Conclusions

Based on data from T4- and FT4-based NBS programs that detect central CH, its incidence is currently estimated at around 1:13,000. Approximately 60% of affected children have central CH as part of CPHD, the others have isolated central CH (≈40%). Most CPHD cases are due to isolated PSIS, a condition of which the etiopathogenesis is still not well understood. In contrast, up to 90% of cases of isolated central CH can be explained by pathogenic variants in *TSHB*, *TRHR*, *IGSF1*, *TBL1X* or *IRS4*.

In the last few years, it has been shown that central CH is a more severe condition than was previously thought, and that in the neonatal period the clinical diagnosis is often missed despite hospital admission because of feeding problems, hypoglycemia and prolonged jaundice. However, with early detection by NBS quickly followed by the right diagnostics and treatment children with central CH, both isolated and CPHD, have an excellent neurodevelopmental prognosis, comparable to unaffected siblings.

## Author Contributions

NZ-S and ASPvT drafted the manuscript. PL and JCN retrieved data for **Figure 1** and **Supplemental Table 2**. All authors contributed to the article and approved the submitted version. ASPvT supervised the whole process.

## Conflict of Interest

The authors declare that the research was conducted in the absence of any commercial or financial relationships that could be construed as a potential conflict of interest.

## Publisher’s Note

All claims expressed in this article are solely those of the authors and do not necessarily represent those of their affiliated organizations, or those of the publisher, the editors and the reviewers. Any product that may be evaluated in this article, or claim that may be made by its manufacturer, is not guaranteed or endorsed by the publisher.
