# Supplementary material for: Diagnosis and Management of Central Congenital Hypothyroidism
Source: Front Endocrinol (Lausanne). 2021 Sep 9;12:686317. doi: 10.3389/fendo.2021.686317 (PMC8458656; doi:10.3389/fendo.2021.686317)
Supplement: Supplementary file 1 [file Table_1.docx]

Supplementary table 1. Reported pathogenic variants in isolated central congenital hypothyroidism. Variant annotations may differ from published variants due to differences between transcripts (indicated in brackets).

*TSHB* (NM_000549.4)

| Type of variant |  |
| --- | --- |
| Missense | c.2T>C, p.(Met1?) (1)  c.94G>A p.(Glu32Lys) (2)  c.145G>A p.(Gly49Arg) (3)  c.313T>C p.(Cys105Arg) (4)  c.323G>A p.(Cys108Tyr) (5) |
| Nonsense | c.94G>T p.(Glu32*) (6)  c.205C>T p.(Gln69*) (4, 7, 8) |
| Frameshift | c.230del p.(Phe77Serfs*6) (9)  c.373del p.(Cys125Valfs*10) (1, 10-13) |
| Splicing site | c.162G>A (exon 2 skip) (5)  c.162+5G>A (exon 2 skip) (14, 15) |
| Deletion | Whole gene deletion (16) |

*TRHR* (NM_003301.5)

| Type of variant | Variant |
| --- | --- |
| Missense | c.242C>G p.(Pro81Arg) (17)  c.353G>A p.(Ala118Thr) (found together with †) (18)  c.392T>C p.(Ile131Thr) (19) |
| Nonsense | c.49C>T p.(Arg17*) (18, 20) |
| Deletion | c.343_352del p.(Ser115_Ala118del) (found together with †) (18) |

*IGSF1* (NM_001170961.1)

| Type of variant | Variant |
| --- | --- |
| Missense | c.1592C>T p.(Ala531Val) (21)  c.1981G>A p.(Gly661Arg) (22, 23)  c.2039T>C p.(Leu680Pro) (24)  c.2267G>A p.(Arg756His) (found together with † †) (23)  c.2309G>A p.(Ser770Asn) (25)  c.2318T>C p.(Leu773Pro) (26)  c.2588C>G p.(Ser863Cys) (21)  c.2588C>T p.(Ser863Phe) (25, 27)  c.2705T>G p.(Met907Arg) (21)  c.2710T>Aa p.(Phe909Ile) (21)  c.2839T>C p.(Cys947Arg) (25)  c.2954T>C p.(Val985Ala) (21)  c.2992G>A p.(Gly998Arg) (21)  c.3127T>C p.(Cys1043Arg) (28)  c.3245T>A p.(Val1082Glu) (29)  c.3416G>T p.(Cys1139Phe) (23)  c.3691T>C p.(Cys1231Gly) (21) |
| Nonsense | c.1847G>A p.(Trp616) (23)  c.1933C>T p.(Gln645*) (29)  c.2008G>T p.(Glu670*) (21)  c.2278G>T p.(Glu760*) (found together with † †) (23)  c.2395C>T p.(Arg799*) (21)  c.2568T>G p.(Tyr856*) (24)  c.2713C>T p.(Gln905*) (30)  c.2931G>A p.(Trp977*) (25)  c.2989C>T p.(Arg997*) (21)  c.3049C>T p.(Gln1017*) (21)  c.3411_3412del, p.(Tyr1137*) (21, 31)  c.3518G>A p.(Trp1173*) (25)  c.3565C>T p.(Arg1189*) (24, 29)  c.3778C>T p.(Gln1260*) (32)  c.3805C>T p.(Arg1269*) (33) |
| Frameshift | c.1137_1138del p.(Asn380Glnfs*6) (34)  c.1705delC p.(Leu569Phefs*16) (21)  c.2248del p.(Glu750Lysfs*28) (25)  c.2283dup p.(Arg762Thrfs*7) (35)  c.2388del p.(Gly797Valfs*4) (23)  c.2422dup p.(His808Profs*14) (21)  c.3251dup p.(Gly1085Trpfs*39) (36)  c.3032delinsTT p.(Gly1101Valfs*14) (21)  c.3517del p.(Trp1173Glyfs*8) (37)  c.3596dup p.(Glu1200Argfs*3) (25) |
| Splicing site | c.2056+1G>A (24, 38)  c.3767-1G>A (21) |
| Deletion/insertion | Whole gene deletion (23-25, 39)  c.2137_2163del p.(Ala713_Lys721del) (25) |

*TBL1X* (NM_005647.3)

| Type of variant | Variant |
| --- | --- |
| Missense | c.1246A>T p.(Asn416Tyr) (40)  c.1249G>A p.(Ala417Thr) (40)  c.1510C>T p.(His504Tyr) (40)  c.1526A>G p.(Tyr509Cys) (40) |
| Synonymous | c.705G>A p.(Val235=) (23) |
| Nonsense | c.1015C>T p.(Arg339*) (41) |
| Frameshift | c.1152del p.(Phe385Leufs*50) (23) |
| Splicing site | c.357+1G>A (23) |
| Deletion | c.1145_1147del p.(Asn382del) (24) |

*IRS4* (NM_003604.2)

| Type of variant | Variant |
| --- | --- |
| Nonsense | c.554C>A p.(Ser185*) (23)  c.643G>T p.(Gly215*) (42)  c.1587_1588insT p.(Arg530Serfs*18) (42)  c.1772dup p.(Lys592Glnfs*12) (42)  c.3161_3165del p.(Cys1054Tyrfs*12) (42) |

**References**

1. Nicholas AK, Jaleel S, Lyons G, Schoenmakers E, Dattani MT, Crowne E, et al. Molecular spectrum of TSHbeta subunit gene defects in central hypothyroidism in the UK and Ireland. **Clin Endocrinol (Oxf)**. 2017;86(3):410-8.

2. Ozhan B, Boz Anlas O, Sarikepe B, Albuz B, Semerci Gunduz N. Congenital Central Hypothyroidism Caused by a Novel Thyroid-Stimulating Hormone-Beta Subunit Gene Mutation in Two Siblings. **J Clin Res Pediatr Endocrinol**. 2017;9(3):278-82.

3. Hayashizaki Y, Hiraoka Y, Endo Y, Miyai K, Matsubara K. Thyroid-stimulating hormone (TSH) deficiency caused by a single base substitution in the CAGYC region of the beta-subunit. **EMBO J**. 1989;8(8):2291-6.

4. Sertedaki A, Papadimitriou A, Voutetakis A, Dracopoulou M, Maniati-Christidi M, Dacou-Voutetakis C. Low TSH congenital hypothyroidism: identification of a novel mutation of the TSH beta-subunit gene in one sporadic case (C85R) and of mutation Q49stop in two siblings with congenital hypothyroidism. **Pediatr Res**. 2002;52(6):935-41.

5. Baquedano MS, Ciaccio M, Dujovne N, Herzovich V, Longueira Y, Warman DM, et al. Two novel mutations of the TSH-beta subunit gene underlying congenital central hypothyroidism undetectable in neonatal TSH screening. **J Clin Endocrinol Metab**. 2010;95(9):E98-103.

6. Dacou-Voutetakis C, Feltquate DM, Drakopoulou M, Kourides IA, Dracopoli NC. Familial hypothyroidism caused by a nonsense mutation in the thyroid-stimulating hormone beta-subunit gene. **Am J Hum Genet**. 1990;46(5):988-93.

7. Vuissoz JM, Deladoey J, Buyukgebiz A, Cemeroglu P, Gex G, Gallati S, et al. New autosomal recessive mutation of the TSH-beta subunit gene causing central isolated hypothyroidism. **J Clin Endocrinol Metab**. 2001;86(9):4468-71.

8. Bonomi M, Proverbio MC, Weber G, Chiumello G, Beck-Peccoz P, Persani L. Hyperplastic pituitary gland, high serum glycoprotein hormone alpha-subunit, and variable circulating thyrotropin (TSH) levels as hallmark of central hypothyroidism due to mutations of the TSH beta gene. **J Clin Endocrinol Metab**. 2001;86(4):1600-4.

9. Morales AE, Shi JD, Wang CY, She JX, Muir A. Novel TSHbeta subunit gene mutation causing congenital central hypothyroidism in a newborn male. **J Pediatr Endocrinol Metab**. 2004;17(3):355-9.

10. Medeiros-Neto G, Herodotou DT, Rajan S, Kommareddi S, de Lacerda L, Sandrini R, et al. A circulating, biologically inactive thyrotropin caused by a mutation in the beta subunit gene. **J Clin Invest**. 1996;97(5):1250-6.

11. Doeker BM, Pfaffle RW, Pohlenz J, Andler W. Congenital central hypothyroidism due to a homozygous mutation in the thyrotropin beta-subunit gene follows an autosomal recessive inheritance. **J Clin Endocrinol Metab**. 1998;83(5):1762-5.

12. McDermott MT, Haugen BR, Black JN, Wood WM, Gordon DF, Ridgway EC. Congenital isolated central hypothyroidism caused by a "hot spot" mutation in the thyrotropin-beta gene. **Thyroid**. 2002;12(12):1141-6.

13. Deladoey J, Vuissoz JM, Domene HM, Malik N, Gruneiro-Papendieck L, Chiesa A, et al. Congenital secondary hypothyroidism due to a mutation C105Vfs114X thyrotropin-beta mutation: genetic study of five unrelated families from Switzerland and Argentina. **Thyroid**. 2003;13(6):553-9.

14. Pohlenz J, Dumitrescu A, Aumann U, Koch G, Melchior R, Prawitt D, et al. Congenital secondary hypothyroidism caused by exon skipping due to a homozygous donor splice site mutation in the TSHbeta-subunit gene. **J Clin Endocrinol Metab**. 2002;87(1):336-9.

15. Borck G, Topaloglu AK, Korsch E, Martine U, Wildhardt G, Onenli-Mungan N, et al. Four new cases of congenital secondary hypothyroidism due to a splice site mutation in the thyrotropin-beta gene: phenotypic variability and founder effect. **J Clin Endocrinol Metab**. 2004;89(8):4136-41.

16. Hermanns P, Couch R, Leonard N, Klotz C, Pohlenz J. A novel deletion in the thyrotropin Beta-subunit gene identified by array comparative genomic hybridization analysis causes central congenital hypothyroidism in a boy originating from Turkey. **Horm Res Paediatr**. 2014;82(3):201-5.

17. Koulouri O, Nicholas AK, Schoenmakers E, Mokrosinski J, Lane F, Cole T, et al. A Novel Thyrotropin-Releasing Hormone Receptor Missense Mutation (P81R) in Central Congenital Hypothyroidism. **J Clin Endocrinol Metab**. 2016;101(3):847-51.

18. Collu R, Tang J, Castagne J, Lagace G, Masson N, Huot C, et al. A novel mechanism for isolated central hypothyroidism: inactivating mutations in the thyrotropin-releasing hormone receptor gene. **J Clin Endocrinol Metab**. 1997;82(5):1561-5.

19. Garcia M, Gonzalez de Buitrago J, Jimenez-Roses M, Pardo L, Hinkle PM, Moreno JC. Central Hypothyroidism Due to a TRHR Mutation Causing Impaired Ligand Affinity and Transactivation of Gq. **J Clin Endocrinol Metab**. 2017;102(7):2433-42.

20. Bonomi M, Busnelli M, Beck-Peccoz P, Costanzo D, Antonica F, Dolci C, et al. A family with complete resistance to thyrotropin-releasing hormone. **N Engl J Med**. 2009;360(7):731-4.

21. Joustra SD, Heinen CA, Schoenmakers N, Bonomi M, Ballieux BE, Turgeon MO, et al. IGSF1 Deficiency: Lessons From an Extensive Case Series and Recommendations for Clinical Management. **J Clin Endocrinol Metab**. 2016;101(4):1627-36.

22. Joustra SD, Roelfsema F, Endert E, Ballieux BE, van Trotsenburg AS, Fliers E, et al. Pituitary Hormone Secretion Profiles in IGSF1 Deficiency Syndrome. **Neuroendocrinology**. 2016;103(3-4):408-16.

23. Naafs JC, Verkerk PH, Fliers E, van Trotsenburg ASP, Zwaveling-Soonawala N. Clinical and genetic characteristics of Dutch children with central congenital hypothyroidism, early detected by neonatal screening. **Eur J Endocrinol**. 2020;183(6):627-36.

24. Sugisawa C, Takamizawa T, Abe K, Hasegawa T, Shiga K, Sugawara H, et al. Genetics of Congenital Isolated TSH Deficiency: Mutation Screening of the Known Causative Genes and a Literature Review. **J Clin Endocrinol Metab**. 2019;104(12):6229-37.

25. Sun Y, Bak B, Schoenmakers N, van Trotsenburg AS, Oostdijk W, Voshol P, et al. Loss-of-function mutations in IGSF1 cause an X-linked syndrome of central hypothyroidism and testicular enlargement. **Nat Genet**. 2012;44(12):1375-81.

26. Roche EF, McGowan A, Koulouri O, Turgeon MO, Nicholas AK, Heffernan E, et al. A novel IGSF1 mutation in a large Irish kindred highlights the need for familial screening in the IGSF1 deficiency syndrome. **Clin Endocrinol (Oxf)**. 2018;89(6):813-23.

27. Heinen CA, Zwaveling-Soonawala N, Fliers E, Turgeon MO, Bernard DJ, van Trotsenburg ASP. A Novel IGSF1 Mutation in a Boy With Short Stature and Hypercholesterolemia: A Case Report. **J Endocr Soc**. 2017;1(6):731-6.

28. Van Hulle S, Craen M, Callewaert B, Joustra S, Oostdijk W, Losekoot M, et al. Delayed Adrenarche may be an Additional Feature of Immunoglobulin Super Family Member 1 Deficiency Syndrome. **J Clin Res Pediatr Endocrinol**. 2016;8(1):86-91.

29. Nakamura A, Bak B, Silander TL, Lam J, Hotsubo T, Yorifuji T, et al. Three novel IGSF1 mutations in four Japanese patients with X-linked congenital central hypothyroidism. **J Clin Endocrinol Metab**. 2013;98(10):E1682-91.

30. Nishigaki S, Hamazaki T, Fujita K, Morikawa S, Tajima T, Shintaku H. A Japanese Family with Central Hypothyroidism Caused by a Novel IGSF1 Mutation. **Thyroid**. 2016;26(12):1701-5.

31. Ghanny S, Zidell A, Pedro H, Joustra SD, Losekoot M, Wit JM, et al. The IGSF1 Deficiency Syndrome May Present with Normal Free T4 Levels, Severe Obesity, or Premature Testicular Growth. **J Clin Res Pediatr Endocrinol**. 2020.

32. Turkkahraman D, Karatas Torun N, Randa NC. A Case of Congenital Central Hypothyroidism Caused by a Novel Variant (Gln1255Ter) in IGSF1 Gene. **J Clin Res Pediatr Endocrinol**. 2020.

33. Papadimitriou A, Papadopoulou A, Kleanthous K, Papadimitriou DT, Papaevangelou V. Hypoprolactinemia as a Clue to Diagnosis of Mild Central Hypothyroidism due to IGSF1 Deficiency. **J Clin Res Pediatr Endocrinol**. 2020;12(2):218-22.

34. Asakura Y, Abe K, Muroya K, Hanakawa J, Oto Y, Narumi S, et al. Combined Growth Hormone and Thyroid-Stimulating Hormone Deficiency in a Japanese Patient with a Novel Frameshift Mutation in IGSF1. **Horm Res Paediatr**. 2015;84(5):349-54.

35. Tenenbaum-Rakover Y, Turgeon MO, London S, Hermanns P, Pohlenz J, Bernard DJ, et al. Familial Central Hypothyroidism Caused by a Novel IGSF1 Gene Mutation. **Thyroid**. 2016;26(12):1693-700.

36. Tajima T, Nakamura A, Ishizu K. A novel mutation of IGSF1 in a Japanese patient of congenital central hypothyroidism without macroorchidism. **Endocr J**. 2013;60(2):245-9.

37. Oguma M, Kobayashi M, Yamazaki M, Yokoyama K, Morikawa S, Yamaguchi T, et al. Two siblings with congenital central hypothyroidism caused by a novel mutation in the IGSF1 gene. **Clin Pediatr Endocrinol**. 2018;27(2):95-100.

38. Yamaguchi T, Hothubo T, Morikawa S, Nakamura A, Mori T, Tajima T. A Japanese patient with congenital central hypothyroidism caused by a novel IGSF1 mutation. **J Pediatr Endocrinol Metab**. 2018;31(3):355-9.

39. Hughes JN, Aubert M, Heatlie J, Gardner A, Gecz J, Morgan T, et al. Identification of an IGSF1-specific deletion in a five-generation pedigree with X-linked Central Hypothyroidism without macroorchidism. **Clin Endocrinol (Oxf)**. 2016;85(4):609-15.

40. Heinen CA, Losekoot M, Sun Y, Watson PJ, Fairall L, Joustra SD, et al. Mutations in TBL1X Are Associated With Central Hypothyroidism. **J Clin Endocrinol Metab**. 2016;101(12):4564-73.

41. Garcia M, Barreda-Bonis AC, Jimenez P, Rabanal I, Ortiz A, Vallespin E, et al. Central Hypothyroidism and Novel Clinical Phenotypes in Hemizygous Truncation of TBL1X. **J Endocr Soc**. 2019;3(1):119-28.

42. Heinen CA, de Vries EM, Alders M, Bikker H, Zwaveling-Soonawala N, van den Akker ELT, et al. Mutations in IRS4 are associated with central hypothyroidism. **J Med Genet**. 2018;55(10):693-700.
